# Oculopalatal tremor following sequential medullary infarcts that did not cause hypertrophic olivary degeneration

**DOI:** 10.1186/s40673-020-00112-2

**Published:** 2020-02-14

**Authors:** Jorge C. Kattah, Rodger J. Elble, Jeffrey De Santo, Aasef G. Shaikh

**Affiliations:** 1 University of Illinois College of Medicicne, Peoria, Illinois USA; 2 Southern Illinois Unbiversity, Carbondale, Illinois USA; 3 University of Illinois, College of Medicine, Peoria, Illinois USA; 4 University Hospitals, Louis Stoke Cleveland Medecial Centter, Case Western Reserve, Cleveland, USA

**Keywords:** Oculopalatal tremor, Olivocerebellar pathway, Medullary infarction, Inferior olivary nucleus

## Abstract

**Background:**

The syndrome of oculopalatal tremor is a known consequence of lesions in the dentate-olivary pathway. Hypertrophic degeneration of the inferior olive is a recognized pathological correlate of these lesions and hypothesized to cause tremorogenic olivary hypersynchrony. However, oculopalatal tremor also occurs in Alexander disease, which produces severe inferior olive degeneration without intervening hypertrophy.

**Methods:**

Serial clinical, imaging, video-oculography and kinematic tremor recording of a patient with oculopalatal and limb tremor.

**Case study:**

We report an unusual presentation of oculopalatal tremor and right upper extremity myorhythmia following sequential right dorsolateral and left anteromedial medullary infarcts directly involving both inferior olives. As in adult Alexander disease, our patient did not have hypertrophic olivary degeneration during 10 years of follow-up.

**Conclusion:**

Contemporary theories have emphasized the role of cerebellar maladaptation in “shaping” oscillations generated elsewhere, the inferior olive in particular. Our patient and published Alexander disease cases illustrate that oculopalatal tremor can occur in the absence of hypertrophic olivary degeneration. Therefore, cerebellar maladaptation to any form of olivary damage may be the critical pathophysiology in producing oculopalatal tremor.

## Introduction

Oculopalatal tremor is a distinct ocular and palatal oscillation that is often found as a late consequence of a lesion within the dentato-olivary pathway [1]. This phenotype of oculopalatal tremor is associated with unilateral or bilateral hypertrophic inferior olive (IO) degeneration that is visible with conventional MRI. Morphologic examination of the IO typically shows vacuolated neurons, astrocytic proliferation, demyelination, dense fibrillary gliosis and eventually complete neuronal death, presumably secondary to trans-synaptic degeneration [2–4]. Synchronized olivary oscillation due to increased gap junctions and maladaptive cerebellar plasticity together are hypothesized to produce oculopalatal tremor [3–5]. Less commonly, oculopalatal tremor occurs in adult-onset medullary Alexander’s disease [6–8] and in GM2-gangliosidosis [9]. Alexander disease produces severe olivary degeneration but without intervening hypertrophy, indicating that hypertrophic olivary degeneration-induced olivo-cerebellar hypersynchrony is not the exclusive mechanism in oculopalatal tremor [6–9]. The pathophysiology of oculopalatal tremor in Alexander disease is unknown. An olivary source of oscillation seems unlikely, and the role of the cerebellum has not been explored. In this report, we describe a rare patient who had two temporally and spatially discrete infarcts – the first one affecting the right dorsolateral medulla and the second affecting the left anteromedial medulla. Both IO were directly, though partially, damaged, but there was no hypertrophic olivary degeneration, despite the development of oculopalatal tremor and right upper extremity myorhythmia. We explored the characteristics of her ocular tremor during voluntary eye movements, seeking insight on the role of the cerebellum on tremorogenesis.

## Methods

The institutional ethics committee at the University of Illinois in Peoria approved the study protocol; while the institutional review board at the Southern Illinois University approved the protocol for limb movement measurements. Prior to enrolling in the study, the patient signed written informed consent forms approved by the respective institutional review boards.

### Eye movement assessment

Head-fixed video-based eye tracker (Otometrics, Natus; spatial resolution = 0.1 deg and temporal resolution = 60 Hz) was used to non-invasively measure horizontal and vertical eye movements in our patient.

The subject sat upright in a chair with head secured in a chinrest. The study protocol involved 1) measurement of eye movements at straight ahead in the absence of visual target; 2) measurement of eye movements at straight ahead and in eccentric orientations in the presence of visual fixation at 5^0^, 10^0^, and 15^0^ to the right and left and up and down; and 3) video head-impulse test to measure the vestibulo-ocular reflex. The data were further processed and analyzed with the commercially available software algorithms called Otosuite (Natus).

For ocular oscillations, the key analyzed variables were the amplitude and frequency of the oscillations. When gaze-evoked nystagmus (or any other form of jerk nystagmus) was present, our assessment focused on the measurement of median slow-phase eye velocity. We identified the epochs of slow-phase (i.e. drifts) in eye positions by excluding the saccades in the eye position waveform. Saccade deletion was performed with a software algorithm, based on eye velocity.

### Tremor assessment

An inertial measurement unit with triaxial accelerometer and gyroscope (Kinesia One; Great Lakes NeuroTechnologies, Cleveland, Ohio) was mounted on the dorsum of the right hand between the second and third metacarpal bones. During the experiment, the patient sat upright in a stationary chair. The trunk was adequately supported to minimize the influence of passive transmitted movements. We recorded tremor for 30 s during posture (forward horizontal extension and the “wing-beating” posture) and movement (finger-nose-finger testing). We subjected the three axes of acceleration and angular velocity to fast Fourier transformation to compute tremor frequency.

## Results

### Clinical presentation

**Fig. 1:**
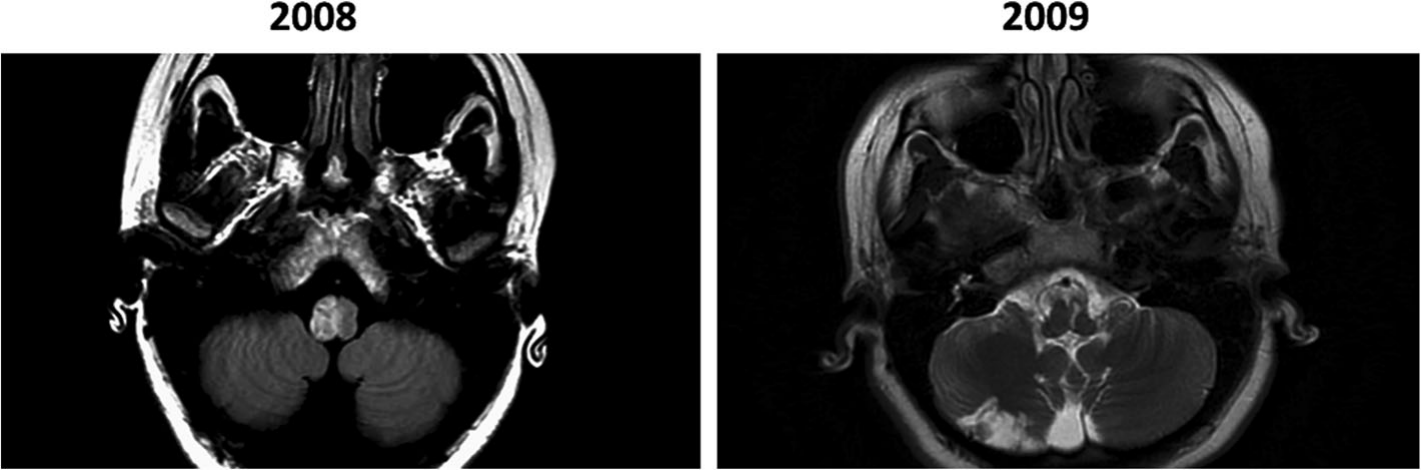
Serial Axial T2 FLAIR MRI. Left panel: increased signal intensity in the right hemi-medulla. Right panel: a head MRI obtained 4 months later revealed a strip of increased signal intensity in the left medial medulla that affects the left medial IO

In December 2008, a 22-year-old woman presented with acute imbalance, nausea and vomiting. On examination, she had right axial lateropulsion, right upper extremity dysmetria and left hemi-hypoesthesia. She also exhibited right ocular lateropulsion right saccade lateropulsion, and horizontal left-beat nystagmus in primary straight-ahead position and in left gaze. The head impulse test was normal. An MRI revealed DWI and T2 signal in the right dorsolateral medulla and faint T2 signal in the right inferior olive (Fig. 1, left panel). CT angiography revealed dissection of the right vertebral artery. Recovery was incomplete, as she had persistent right face and left body sensory loss and mild incoordination of gait. Four months later, she developed acute weakness of the right side, increased imbalance, and new onset diplopia and oscillopsia. Subsequent examination revealed new right hemiparesis in addition to previous sensory and coordination deficits. She also had a horizontal/torsional right beating nystagmus that increased in right gaze; left gaze was associated with left beating nystagmus. Right horizontal saccades were hypermetric, and horizontal pursuit was bilaterally saccadic. She had decreased optokinetic nystagmus gain bilaterally (0.52 and 0.54). Bithermal caloric testing and head impulse tests were normal bilaterally. Ocular cross cover test detected a new large skew deviation with a 12-prism diopter right hypertropia. In a follow-up visit 6 weeks later, her neurological examination was unchanged except for the additional finding of pendular vertical eye oscillations and bilateral palatal tremor.

An MRI 1 month after her second stroke revealed an infarct extending from the left medullary pyramid posteriorly to the posterior surface of the medulla (Fig. 1, left panel). This infarct appeared to involve the medial left inferior olive, but there was no olivary hypertrophy. We found dissection of the left vertebral artery with CT angiography. Subsequent MRI scans at intervals of 2, 3, 8, and 10 years did not reveal additional infarcts or inferior olive hypertrophy.

Ten years after the very first event (May 2018), at the time of objective ocular motor assessment reported in this study, she had diplopia that was corrected with a right six-diopter base-down prism but still had mild right head tilt.

The general neurologic examination revealed a right hemiparesis with circumduction of the right leg, and she exhibited moderate right-sided extremity ataxia, although this was difficult to assess due to hemiparesis. She also exhibited right side hyperalgesias and impaired sensation to the sharp objects in the left hemi body. She had palatal tremor with a frequency of 1.8 Hz (Video), as measured by counting the movements in the video frames. She had a proximal tremor in her right upper limb during posture, movement but not at rest. The tremor was greatest in the wing-beating posture.


**Additional file 1: Video 1**. The first section shows a dissociated vertical pendular oscillation with a subtle torsional component noted during fixation at a target straight ahead. The second section shows the effect of fixation block. Note the conjugate horizontal, jerk right-beat nystagmus. The third section shows a rhythmic movement of the soft palate and uvula.


#### Quantitative ocular motor and tremor assessment

**Fig. 2:**
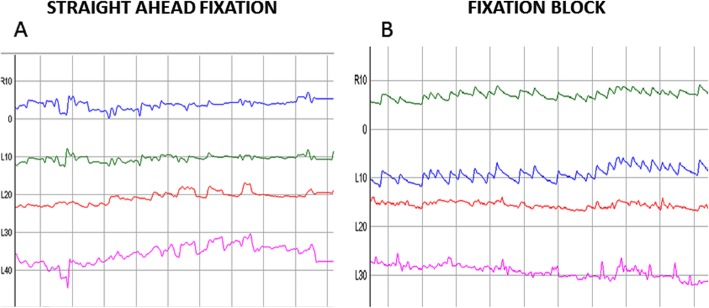
Video-oculography: Eye movement recording during straight-ahead fixation revealed a 2 Hz pendular vertical oscillation. The pendular oscillation was binocularly dissociated; the amplitude was greater in the right eye (left panel). Eye movement recorded with fixation block (right panel): note a horizontal, jerk right-beat nystagmus that replaced the vertical oscillation. These findings have been unchanged for 10 years

**Fig. 3:**
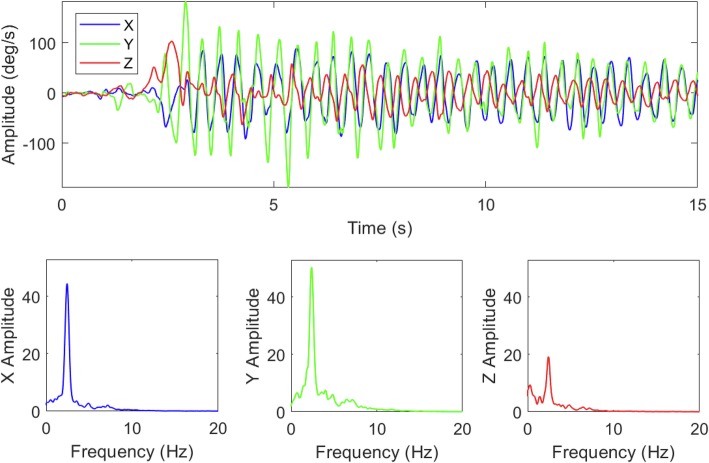
Right upper extremity tremor was recorded with a triaxial gyroscopic motion transducer mounted on the dorsal surface of the hand, between the second and third metacarpal bones, just proximal to the metacarpophalangeal joints. The X, Y, and Z-axes of the transducer were oriented laterally, axially and perpendicular to hand, measuring pitch, roll and yaw angular velocity (deg/s) of the hand. The recording began with the right upper limb extended horizontally in front of the patient. At two seconds, the patient flexed the elbow about 100 deg, bringing her upper limb into the so-called wing-beating posture, with her hand positioned about 10 cm anterior to her chin. Tremor increased greatly when the wing beating posture was assumed (upper graph). The amplitude spectra shown in the lower three graphs are the distributions of X, Y and X root mean square angular velocity distributed over frequency (Hz). The spectra demonstrate a finely tuned oscillation at 2.4 Hz

We performed quantitative ocular motor assessment in May 2018, and quantitative tremor analysis occurred in September 2018. Eye movement recording during straight-ahead fixation revealed a 2 Hz pendular vertical oscillation. The pendular oscillation was binocularly dissociated; the amplitude was greater in the right eye (Fig. 2, Video). She had impaired bi-directional horizontal and vertical pursuit, and rightward saccades were hypermetric with normal velocity and latency. With fixation block, she exhibited a straight-ahead horizontal right-beating nystagmus with a slow phase velocity of 7 deg/sec (Fig. 3B, Video 1 second section). She had modestly decreased horizontal left (0.7) compared to normal right horizontal (0.9) vestibulo-ocular reflex gain (normal range: 0.8–1.0). Occasional square wave jerks were present. Vestibulo-ocular reflex cancellation was normal. Her tremor was recorded with a triaxial accelerometer and gyroscope mounted on the dorsum of the right hand. Spectral analysis revealed a tremor frequency of 2.4 Hz during posture and movement.

## Discussion

The syndrome of oculopalatal tremor in dentato-olivary lesions involves a well-defined anatomic pathway and the unique pathology of hypertrophic olivary degeneration. The inferior olive is widely proposed as the primary source of oscillation in this syndrome. Olivary hypersynchrony has been thought to cause maladaptive cerebellar plasticity that smooths and amplifies the olivary oscillations [5]. Our patient had ischemic damage to both inferior olives and the midline medulla where olivary fibers cross en route to the contralateral cerebellum. Given the extent of her infarcts and absence of olivary “hypertrophy” in the MRI, we conclude that our patient did not have pseudohypertrophic olivary degeneration. Nevertheless, she developed bilateral oculopalatal tremor. Adult-onset Alexander disease also causes oculopalatal tremor and severe olivary degeneration in the absence of olivary hypertrophy, making the IO an unlikely source of tremorogenic oscillation [6–8]. In addition, adult onset GM2 gangliosidosis in a 71-year old patient caused oculopalatal tremor in the absence of olivary hypertrophy.

Cerebellar maladaptation is hypothesized to facilitate coarse, irregular oscillations in patients with typical oculopalatal tremor [5]. This hypothesis of oculopalatal tremor emphasizes a dual oscillator model in which primary oscillation originates in the IO and is facilitated by secondary plasticity in the cerebellum [5]. Data from our patient suggest that maladaptive cerebellar plasticity could underlie oculopalatal tremor even when the IO is not the primary source of oscillation. A prediction of this hypothesis is that functional or structural alterations in the cerebellum will modulate the characteristics of the ocular oscillation waveforms. Our patient had gaze-evoked nystagmus shifted null secondary to a right dorsolateral medullary infarct, which appeared to involve a portion of the right IO. A subsequent lesion in the left ventromedial medulla probably also affected IO input to cerebellum. We hypothesize that this disruption of olivary input altered cerebellar function in a way that oculopalatal tremor and right upper extremity myorhythmia ultimately developed.

Several theories have been forthcoming to explain the ocular oscillations. Kim suggested that oscillations resulted from an asymmetry of the input between the right and left IO, leading to changes in paramedian tract neurons that mediate vertical gaze [10]. The ocular oscillation in our case bears significant similarity to the oculopalatal tremor reported by Jang and Borruat with vertical dissociated nystagmus [11]. In our case, however, with fixation block, a horizontal right-beat nystagmus replaced the vertical oscillation, and this nystagmus failed to adapt in serial follow-up over a decade. In addition, she also had right gaze holding failure. We hypothesize that the cerebellum is continuously engaged in a permanent regulation of the ocular tremor, and thus unable to perform its constructive (normal adaptive) role to suppress the nystagmus over time and to maintain lateral gaze position.

Oculopalatal tremor has been regarded as a form of myorhythmia (< 4-Hz tremor), [12] which characteristically occurs weeks to months after brainstem and cerebellar strokes, with or without hypertrophic degeneration of the IO [13]. Our patient developed action myorhythmia in her right upper extremity. Previous reports of IO infarction [14, 15] and experimental destruction [16] did not describe myorhythmia or oculopalatal tremor, but these patients and laboratory animals may not have been followed long enough for sufficient maladaptive plasticity to occur.

Data from our patient and published Alexander disease cases conflict with the notion that oculopalatal tremor exclusively stems from abnormal olivary hyper-synchrony, but these data are compatible with the hypothesis that the olivocerebellar pathway normally functions to suppress abnormal or latent rhythmicity (e.g., myorhythmia) in brainstem networks [2, 17–19]. Recordings from unanesthetized monkeys and mice suggest that the normal olivocerebellar pathway is resistant to sustained oscillation, and the putative role of olivary oscillation in tremor is based on the questionably-relevant harmaline model and has no direct experimental support [20]. It is possible that olivary destruction of any type can lead to maladaptive cerebellar plasticity conducive to oculopalatal tremor and other forms of myorhythmia.

## Data Availability

All data in this report is available for review upon request.

## Abbreviation

IO: Inferior olive
